# TIMS^2^Rescore: A Data Dependent Acquisition-Parallel Accumulation and Serial Fragmentation-Optimized Data-Driven Rescoring Pipeline Based on MS^2^Rescore

**DOI:** 10.1021/acs.jproteome.4c00609

**Published:** 2025-02-07

**Authors:** Arthur Declercq, Robbe Devreese, Jonas Scheid, Caroline Jachmann, Tim Van Den Bossche, Annica Preikschat, David Gomez-Zepeda, Jeewan Babu Rijal, Aurélie Hirschler, Jonathan R Krieger, Tharan Srikumar, George Rosenberger, Claudia Martelli, Dennis Trede, Christine Carapito, Stefan Tenzer, Juliane S Walz, Sven Degroeve, Robbin Bouwmeester, Lennart Martens, Ralf Gabriels

**Affiliations:** 1 VIB-UGent Center for Medical Biotechnology, VIB, Ghent 9052, Belgium; 2 Department of Biomolecular Medicine, Ghent University, Ghent 9052, Belgium; 3 Department of Peptide-based Immunotherapy, Institute of Immunology, University and University Hospital Tübingen, Tübingen 72076, Germany; 4 Cluster of Excellence iFIT (ECX2180) Image-Guided and Functionally Instructed Tumor Therapies, University of Tuebingen, Tuebingen 72076, Germany; 5 Quantitative Biology Center (QBiC), University of Tübingen, Tübingen 72076, Germany; 6 Institute of Immunology, University Medical Center of the Johannes-Gutenberg University, Mainz 55131, Germany; 7 Helmholtz Institute for Translational Oncology Mainz (HI-TRON Mainz) − A Helmholtz Institute of the DKFZ, Mainz 55131, Germany; 8 German Cancer Research Center (DKFZ) Heidelberg, Division 191 & Immunopeptidomics Platform, Heidelberg 69120, Germany; 9 BioOrganic Mass Spectrometry Laboratory (LSMBO), IPHC UMR 7178, University of Strasbourg, CNRS, ProFI FR2048, Strasbourg 67087, France; 10 Bruker Ltd., Milton, Ontario L9T 6P4, Canada; 11 Bruker Switzerland AG, Faellanden 8117, Switzerland; 12 Bruker Daltonics GmbH & Co. KG, Bremen 28359, Germany; 13 Research Center for Immunotherapy (FZI), University Medical Center of the Johannes-Gutenberg University, Mainz 55131, Germany; 14 Clinical Collaboration Unit Translational Immunology, Department of Internal Medicine, University Hospital Tuebingen, Tuebingen 72076, Germany; 15 German Cancer Consortium (DKTK) and German Cancer Research Center (DKFZ), partner site Tübingen, Tübingen 72076, Germany

**Keywords:** proteomics, mass spectrometry, DDA-PASEF, timsTOF, machine learning, rescoring, peptide identification

## Abstract

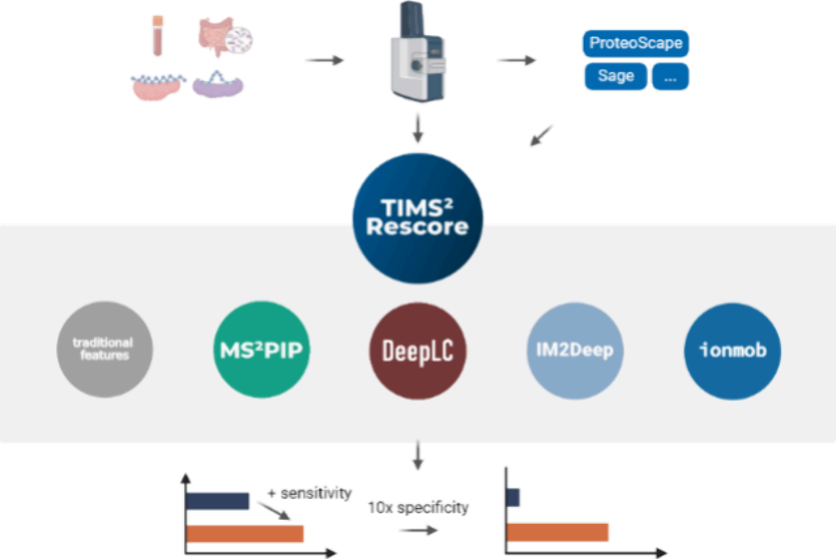

The high throughput analysis of proteins with mass spectrometry (MS) is highly valuable for understanding human biology, discovering disease biomarkers, identifying therapeutic targets, and exploring pathogen interactions. To achieve these goals, specialized proteomics subfields, including plasma proteomics, immunopeptidomics, and metaproteomics, must tackle specific analytical challenges, such as an increased identification ambiguity compared to routine proteomics experiments. Technical advancements in MS instrumentation can mitigate these issues by acquiring more discerning information at higher sensitivity levels. This is exemplified by the incorporation of ion mobility and parallel accumulation and serial fragmentation (PASEF) technologies in timsTOF instruments. In addition, AI-based bioinformatics solutions can help overcome ambiguity issues by integrating more data into the identification workflow. Here, we introduce TIMS^2^Rescore, a data-driven rescoring workflow optimized for DDA-PASEF data from timsTOF instruments. This platform includes new timsTOF MS^2^PIP spectrum prediction models and IM2Deep, a new deep learning-based peptide ion mobility predictor. Furthermore, to fully streamline data throughput, TIMS^2^Rescore directly accepts Bruker raw mass spectrometry data and search results from ProteoScape and many other search engines, including Sage and PEAKS. We showcase TIMS^2^Rescore performance on plasma proteomics, immunopeptidomics (HLA class I and II), and metaproteomics data sets. TIMS^2^Rescore is open-source and freely available at https://github.com/compomics/tims2rescore.

## Introduction

Proteomics is an invaluable tool for understanding human biology, facilitating the discovery of new disease biomarkers, identifying potential therapeutic targets, and exploring interactions with pathogens or microorganisms.^1−4^ Various proteomics subfields have emerged that address specific challenges. For example, plasma proteomics tackles the vast dynamic range of protein abundances, immunopeptidomics deals with the nontryptic nature of immunopeptides combined with varying lengths, i.e. class I 8–12 amino acids and class II 12–25 amino acids, and metaproteomics must address the complexity of multiple species that have highly similar tryptic peptides within each sample.^4−6^ These challenges all contribute to higher identification ambiguity, stemming from a higher complexity in the acquired data, a larger and more diverse peptide search space, high sequence similarities, or all of the above.^7^ To overcome these issues, highly sensitive yet specific peptide spectrum identification strategies are required.

Artificial intelligence (AI) has undeniably transformed many research fields, including computational proteomics. AI allows us to predict analyte behavior for almost every step in the liquid chromatography - ion mobility - tandem mass spectrometry (LC-IM-MS/MS) pipeline,^8,9^ from peptide retention times to the intensities of peptide fragmentation spectra. Indeed, machine learning tools such as DeepLC^10^ and MS^2^PIP^11^ can predict these values accurately and precisely. Recently, such predictors have been shown to be highly powerful means to enhance identification performance through data-driven rescoring.^12−14^ In this approach, predictions for each candidate peptide-spectrum match (PSM) are first compared to the observed data. These comparison values are then fed to a semisupervised machine learning algorithm, which rescores the PSMs based on all available information. Data-driven rescoring algorithms such as MS^2^Rescore^12^ have been shown to substantially increase identification sensitivity and specificity while maintaining a proper statistical false discovery rate (FDR) control. For example, for immunopeptidomics data, increases in identification rate of over 35% at 1% FDR have been reported.^15−17^

Technical advances to LC-IM-MS/MS instruments, exemplified by timsTOF instruments, have also greatly improved identification performance in challenging proteomics subfields. While standard LC-MS/MS systems rely solely on the LC setup and the quadrupole for peptide separation preceding fragmentation, timsTOF instruments incorporate IM for additional ion separation in the gas phase based on collisional cross section. Moreover, due to the parallel accumulation and serial fragmentation (PASEF) technology, precursor ions are accumulated in the TIMS tunnel before being released sequentially, leading to a much-improved sensitivity.^18^ This higher sensitivity is particularly beneficial for detecting low-abundance ions, common in immunopeptidomics or plasma proteomics. Indeed, timsTOF instruments have been shown to substantially boost identification rates for both class I and class II immunopeptides,^6,19,20^ and to allow for much broader plasma protein profiling.^21^

We here present TIMS^2^Rescore, a new version of our data-driven rescoring platform MS^2^Rescore, optimized for data dependent acquisition PASEF (DDA-PASEF) data from timsTOF instruments. First, we have trained new timsTOF-compatible MS^2^PIP spectrum prediction models, which were subsequently validated on plasma proteomics, immunopeptidomics, and metaproteomics data sets. Second, to optimally leverage the additional information provided in the IM dimension, we have developed IM2Deep, a deep learning-based peptide collisional cross section (CCS) predictor that uses a similar architecture to our state-of-the-art retention time predictor, DeepLC. As a result, IM2Deep is able to accurately predict CCS values for both unmodified as well as modified peptides, even if those modifications were not seen during training. Third, for an optimal software integration, TIMS^2^Rescore directly accepts Bruker raw mass spectrometry data and search results from Bruker ProteoScape and various other search engines, including Sage and PEAKS. Finally, we evaluated the full TIMS^2^Rescore workflow, including MS^2^PIP, IM2Deep, and DeepLC, on data sets from plasma proteomics, immunopeptidomics and metaproteomics experiments. In all three cases, TIMS^2^Rescore shows substantial increases in identification performance. Thus, TIMS^2^Rescore will enable researchers to obtain a broader and more confident peptide and protein identification coverage for a large variety of applications.

## Methods

### Specialized MS^2^PIP models for timsTOF fragmentation

A new model (timsTOF 2024) was trained following the procedures described in the 2023 MS^2^PIP publication^19^. The trypsin, elastase, and class I immunopeptide data that was used to train the original timsTOF 2023 model (PXD046535, PXD040385 and PXD046543) was now supplemented with class II immunopeptides retrieved from Hoenisch Gravel et al.^20^ (PXD038782). The 505,289 highest scoring peptidoforms across all data sets were retained–considering precursor charge as part of the peptidoform. These were then further separated into a training set (480,024 peptidoforms) and a test set (25,265 peptidoforms) using a stratified division based on the source data set, ensuring sufficient peptides from each peptide type, i.e. class I, class II, trypsin-digested, and elastase-digested peptides in each subset. All processed data is available on Zenodo at 10.5281/zenodo.11277943. The training set was used to train XGBoost (v1.7.2)^22^ models for singly charged b- and y-ions. The Hyperopt (v0.2.7) package was used for hyperparameter optimization, employing a 5-fold cross-validation. The full hyperparameter optimizations were logged with Weights and Biases. For model evaluation, not only the highest scoring test peptidoforms, but all PSMs (193,400) for the test peptidoforms (25,265) were retrieved from the search data. For each PSM, the observed b- and y- ion intensities were retrieved, and predictions were made with the 2021 Orbitrap HCD (higher energy collisional dissociation) model, the timsTOF 2023 model, and the newly trained timsTOF 2024 model. Furthermore, the observed intensities for PSMs coming from the same peptidoforms were also correlated to each other in Pearson correlation coefficient (PCC) to measure the inherent variability within experimental data, providing an estimate of the accuracy that can be achieved with prediction models.

### IM2Deep Collisional Cross Section Prediction

The deep learning architecture of IM2Deep mirrors that of DeepLC and is described in detail in Bouwmeester et al.^10^ Briefly, IM2Deep employs a convolutional architecture with four distinct paths, through which each encoded peptide is propagated. Three paths utilize convolutional and maximum pooling layers to capture local structures. These paths handle atomic composition of amino acids, atomic composition of diamino acids and one-hot encoding for unmodified amino acids. A fourth path passes on global features through fully connected layers, including length, total atomic composition, and composition at specific positions.

The sole difference between IM2Deep and DeepLC is the addition of five features to the global feature matrix: (1) the relative frequency of histidine within the peptide sequence, (2) the relative frequency of bulky amino acids (F, W, Y), (3) the relative frequency of acidic amino acids (D, E), (4) the relative frequency of lysine and arginine (K, R), and (5) the charge state of the peptide ion. These features were included because, as has been shown earlier, groups of amino acids with similar physicochemical properties can have a similar impact on the CCS and are thus grouped.^23^ The model combines the results from all paths through flattening and concatenation, providing an input for six connected dense layers in the final combined path, which outputs the predicted CCS value.

To train and evaluate IM2Deep on its ability to generalize its predictions on modifications and amino acids unseen during training, two data sets were combined into one large data set. The first data set, described by Meier et al.^23^ consists of 718,917 unique combinations of peptide sequence, charge state and, when applicable, modifications (limited to methionine oxidation, cysteine carbamidomethylation and N-terminal acetylation). The second data set, described by Will et al.,^24^ comprises 5,202 unique peptidoform-charge state combinations, and contains a wider variety of modifications. In this data set, a distinction is also made between symmetrical and asymmetrical arginine dimethylation. However, as IM2Deep is not able to distinguish between isomeric differences in peptides, we used the mean CCS value of these isomers as the CCS value for the dimethylated peptide-charge state pair. To account for experimental drifts in the measurements of CCS values between the two data sets, we performed an alignment by calculating the linear offset (*y = ax + b*) between overlapping peptide-charge pair states in the two data sets, according to Meier et al.^23^ and Prianichnikov et al.^25^ Only unique peptidoform-charge states were retained in the data set, and the mean value of overlapping pairs, after alignment, was used to train and evaluate the models. Trained models were initialized with random weights drawn from a normal distribution (μ = 0.0 and σ = 1.0). A single NVIDIA Geforce RTX 4090 graphic card was used for training, which lasted for maximally 300 epochs, with early stopping on a validation set to prevent overfitting.

To allow the ion mobility dimension to be used for rescoring TIMS data, IM2Deep was implemented as a feature generator within TIMS^2^Rescore. The final IM2Deep model shipped with TIMS^2^Rescore was trained and evaluated (89.1% training, 0.9% validation, 10% test, with no overlap in peptidoforms) on the data set described above, in combination with an immunopeptidomics data set^20^ which consists of 437,479 unique (modified) peptide-charge pairs, most of which are nontryptic. Before merging, the immunopeptidomics data set was aligned to the original data set using a linear offset between overlapping precursors.

The rescoring features generated by IM2Deep include the observed and predicted CCS values, alongside the absolute and percentual error between the observed and predicted CCS. Before computing these features, the predictions are calibrated to the observed CCS range by calculating the linear offset between the CCS values of a reference data set and the overlapping precursors in the 75% most confidently identified precursors at 1% FDR before rescoring.

### Data-Driven Rescoring

The full TIMS^2^Rescore pipeline, with the new MS^2^PIP and IM2Deep models, was evaluated on unseen plasma proteomics, immunopeptidomics, and metaproteomics data sets. The plasma proteomics data, provided by Bruker, and the class II immunopeptidomics data were generated in-house (see Supplementary Methods) and deposited to the ProteomeXchange Consortium via the PRIDE (PXD053748) and jPOSTrepo (JPST003195, PXD053756) partner repositories, respectively. The immunopeptidomics class I data was obtained from the same large-scale immunopeptidomics study from Gravnel et al.^20^ (PXD038782) that provided the class II data used for MS^2^PIP training. Lastly, all metaproteomics samples were acquired from the CAMPI study^4^ (PXD023217). All data was searched using Sage (v0.14.3)^26^ with 10 ppm precursor and fragment tolerances and a maximum of two variable modifications. In all four searches, oxidation of methionine was considered as variable modification. For both immunopeptidomics data sets, carbamidomethylation of C was considered as variable modification as well. For the plasma and metaproteomics data sets, carbamidomethylation of C was set as fixed modification and acetylation of the protein N-termini was added as additional variable modification. For the immunopeptidomics searches, no cleavage rule was used and peptide lengths set to 8–25 for class I and 8–30 for class II. The plasma and metaproteomics data sets were searched with trypsin as cleavage rule with a restriction for proline and peptide lengths were set to 8–50 for plasma and 8–30 for metaproteomics. Aside from the metaproteomics data, where the custom sequence database was used that was published alongside the MS data^4^, all data sets were searched using the Swiss-Prot canonical human proteome (UP000005640, 20,597 entries, downloaded March 2024). Similarly, we downloaded *Escherichia coli* data from PXD028735^27^ for an entrapment experiment, which was searched with Sage with the same settings as the plasma data set. The database consisted of *E. coli* proteins (UP000000625, 4401 entries, downloaded November 2024), supplemented with either *H. sapiens* (UP000005640, 20,597 entries, downloaded March 2024) or *Pyrococcus furiosus (UP000001013,* 2044 entries, downloaded November 2024). Furthermore, as entrapment experiments with human sequences are difficult due to human-derived contaminants, we supplemented both with an extensive contaminant database as described in Frankenfield et al.^28^ All data was rescored using TIMS^2^Rescore, with all feature generators enabled. Furthermore, the plasma data set was also rescored with every feature generator separately, combined with the traditional PSM file features as provided by the search engine.

## Results

### TIMS^2^Rescore: Data-Driven Rescoring Tailored to timsTOF Instruments

TIMS^2^Rescore is built on top of the data-driven rescoring framework MS^2^Rescore. Several improvements were made to create a streamlined rescoring workflow that is fully tailored to DDA-PASEF data from timsTOF instruments. First, to drastically speed up reading of large spectrum files, we integrated the Rust-based mzdata file readers for the MGF and mzML file formats (https://github.com/mobiusklein/mzdata). Moreover, we have implemented direct support for Bruker TDF and mini-TDF raw formats, using the TimsRust package (https://github.com/mannlabs/timsrust), allowing users to avoid long data conversion steps altogether. Second, as support for DDA-PASEF data was recently added to the ultrafast search engine Sage,^26^ we added direct support for Sage PSM files in TIMS^2^Rescore, along with support for PSM files from the Bruker ProteoScape search environment. Third, a new set of default parameters optimized for rescoring DDA-PASEF data are made available. Combined with the new prediction models outlined below, these features significantly improve the ease of use and computational performance for rescoring timsTOF data.

### MS^2^PIP Prediction Models

**Figure 1:**
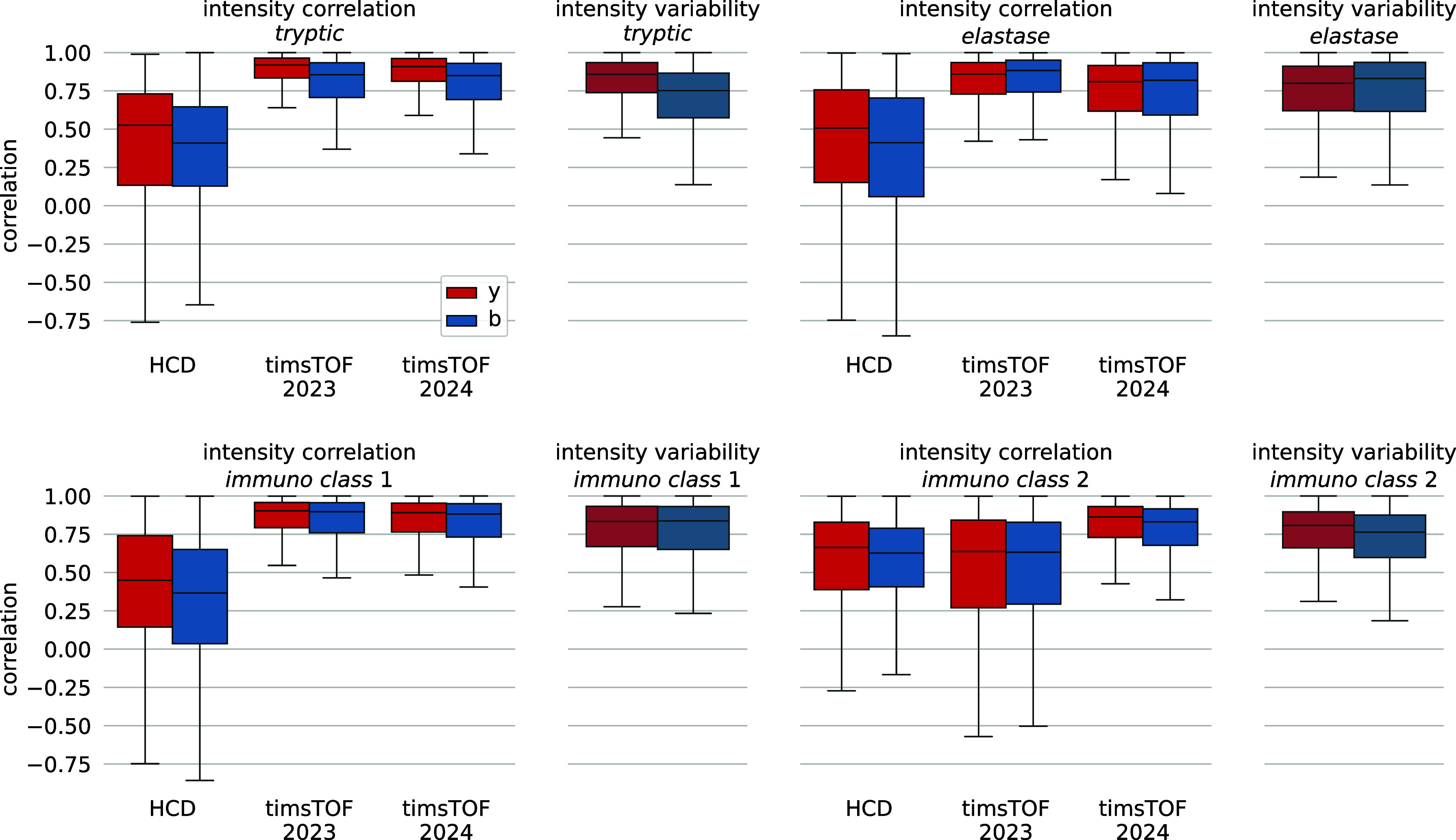
Boxplots showing Pearson correlation coefficients between predicted and observed intensities for different models. The correlation between observed intensities across different spectra for the same peptidoform ions are shown in muted colors. Four different peptide subgroups are analyzed: (a) tryptic peptides, (b) elastase digested peptides, (c) class I immunopeptides, and (d) class II immunopeptides. All correlations were calculated on intensity values normalized to the total-ion-current and log_2_-transformed.

We, and others, have previously shown that different fragmentation methods can heavily alter peptide MS2 spectra.^29,30^ We therefore trained new MS^2^PIP models that can accurately predict peak intensities for timsTOF acquired peptides. In 2023, we trained new timsTOF-compatible models on tryptic peptides, elastase digested peptides, and class I immunopeptides, which we subsequently used to boost immunopeptides identification performance through rescoring.^19^ While this model performed well for data sets similar to the aforementioned training peptides (median PCC 0.89, 0.89, 0.87 for tryptic, class I immunopeptides and elastase peptides, respectively), the performance for class II immunopeptides was significantly lower (median PCC 0.64) and was even outperformed by the model for Orbitrap HCD spectra (Figure 1). This could be due to the generally longer peptides which were not yet seen during training, as class I immunopeptides and elastase peptides are generally shorter. To overcome these issues, we have trained a new MS^2^PIP model on data that was supplemented with a large amount of class II immunopeptides. The newly trained model performs comparable to the previous timsTOF model for tryptic (0.89 median PCC) and class I immunopeptides (0.88 median PCC), performs slightly worse for elastase digested peptides (0.81 median PCC), but performs drastically better for the class II immunopeptides (0.85 median PCC) (Figure 1). While the performance drops slightly for elastase peptides, the prediction accuracy is still close to the expected intensity variability seen in timsTOF spectra, which is also the case for all other peptide types. This drop could potentially be attributed to the lower amount of training peptides for elastase relative to the newly added class II immunopeptides (35,126 vs 232,798 peptides, respectively). When examining peptides with a higher variation in observed intensities across different spectra, we can see that the MS^2^PIP predictions still approximate the median intensity, highlighting the robustness of the newly trained model (Supplementary Figure S1). Overall, the 2024 timsTOF model performs either similar to, or better than, the timsTOF 2023 model depending on the peptide type. All median PCCs are listed in Supplementary Table S1.

### IM2Deep

#### IM2Deep Performance on Modified Peptides

To assess the CCS prediction ability of IM2Deep across a variety of differently modified peptides, we systematically evaluated its performance on all twenty-one modifications within the combined data set. Our approach involved training and optimizing twenty-one individual IM2Deep models, each exclusively trained on peptides not carrying one specific modification. These models were subsequently tested on peptides that do carry the excluded modification. Furthermore, we created two test scenarios: One where the excluded modification was encoded, and another where it was ignored. By comparing the prediction performance for both test scenarios, we gauged the ability of IM2Deep to predict the CCS for peptides with modifications unseen during training. This comparison aims to measure the improvement provided by IM2Deep over a basic approach that simply disregards the presence of the modification.

**Figure 2:**
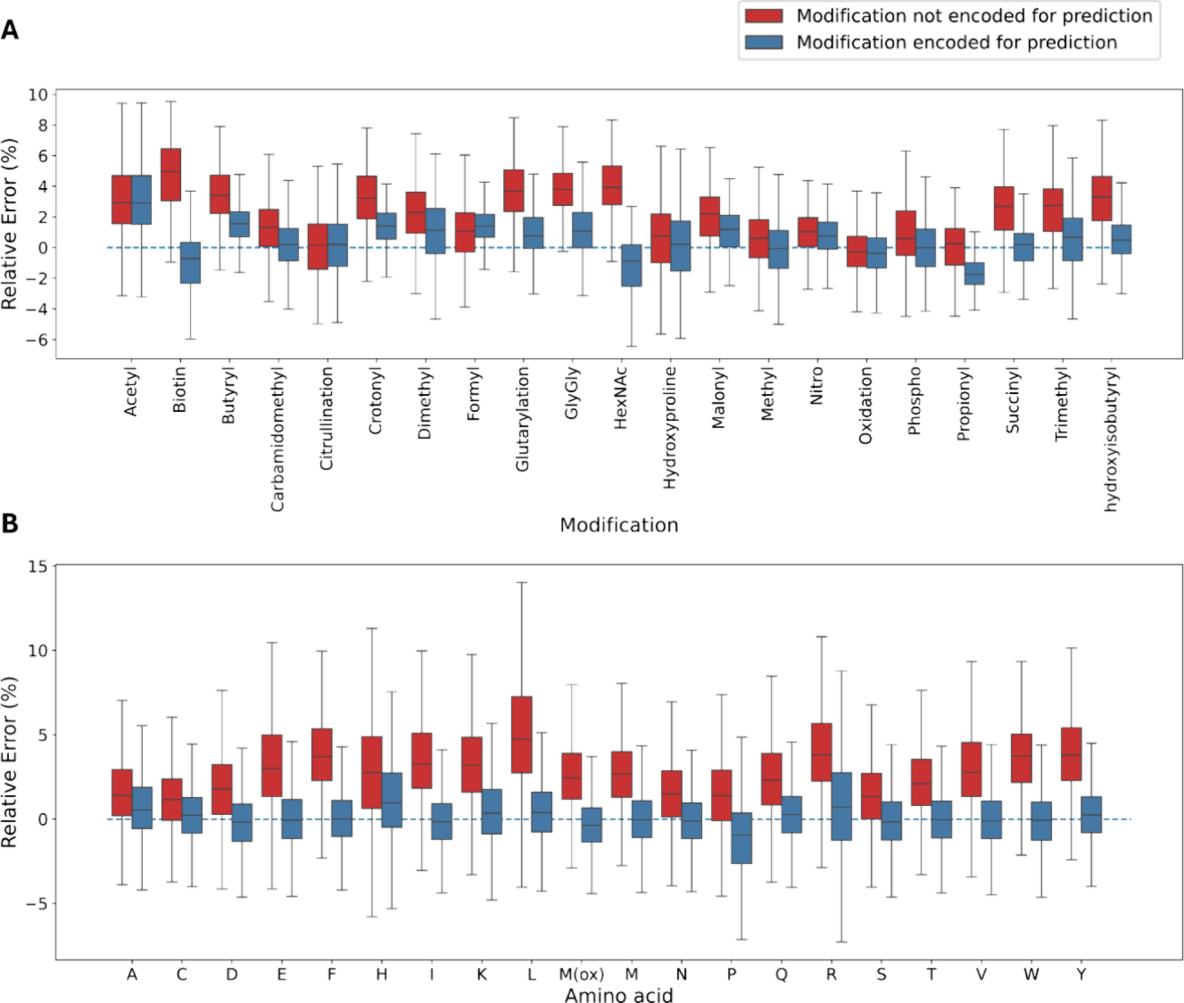
IM2Deep evaluation approach involved training individual models, each exclusively trained on peptides not carrying a specific modification/not containing a specific amino acid. The box plots show the IM2Deep prediction errors for peptides with modifications (A) and amino acids (B) that were not seen during training each of the respective models. Horizontal axis represents the excluded modifications/amino acids, while the vertical axis depicts the absolute error between the observed and predicted CCS when the modification/amino acid was either not encoded (red) or encoded during predictions (blue). These results indicate that IM2Deep generalizes well across modifications and amino acids, even if these were not seen during training. Note that peptides containing cysteine have a fixed carbamidomethyl modification.

**Figure 3:**
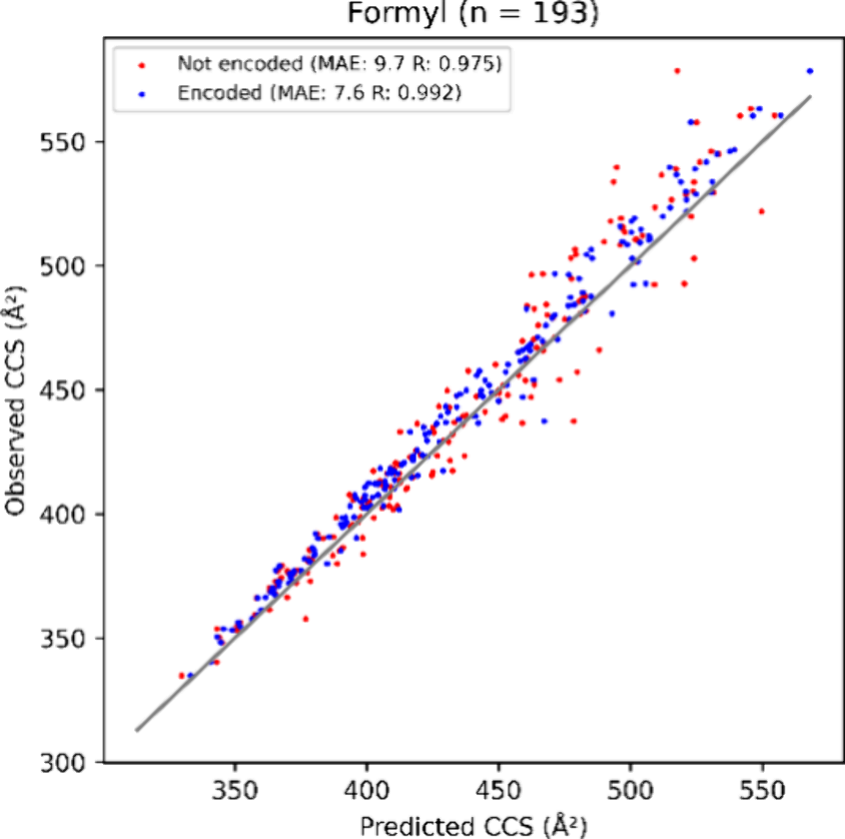
Scatterplot illustrating the performance of the IM2Deep model not trained on formylated peptides in predicting CCS for formylated peptides. The model was evaluated both with the modification encoded (blue) and ignored (red). Besides an improvement in MAE, an increase in Pearson correlation coefficient is also seen. Variable n denotes the total number of formylated peptides.

The prediction errors for each of the omitted modifications during training (Figure 2A) show an overall performance improvement when modifications are encoded during prediction. The observed reductions in mean absolute error (MAE) stem from IM2Deep’s accurate prediction of the CCS shift induced by the respective modifications, even though they were unseen during training. In addition to the reduced MAE, a general improvement in PCC is also observed. For example, in the case of formyl, an increase in PCC from 0.975 to 0.992 can be observed when encoding the modification in the test set (Figure 3, Supplementary Figure S2). This indicates that IM2Deep models not only learned the overall shift in CCS caused by modifications, but also captured how this shift depends on the specific context of the modification within each peptide.

Furthermore, no discernible relationship could be observed between performance improvements and the Tanimoto similarity with the closest related modification, suggesting that IM2Deep’s generalization capacity is not dependent on chemically similar modifications in the training data (Supplementary Figure S3). The training set sizes were also comparable across models, except for those sets omitting carbamidomethylation and oxidation, which were represented by a notably smaller number of peptides, and no clear relationship to performance improvement was observed (Supplementary Figures S4 and S5).

In a second evaluation procedure, the same data set was used to train 19 distinct IM2Deep models, each exclusively trained on peptides that lack a specific amino acid. Subsequently, each model underwent evaluation on peptides that did contain the amino acid excluded during training. Here too, two distinct test sets were generated from these remaining peptides: one where the excluded amino acid was encoded with its actual composition, and another where its composition was substituted with that of glycine. In this analysis, all peptides carrying modifications apart from methionine oxidation and cysteine carbamidomethylation were removed to focus on the performance on unseen amino acids. We demonstrate that encoding an amino acid as its own entity rather than as glycine lowers the MAE and increases the PCC for most amino acids (Figure 2B, Supplementary Figure S5). Note that the diminished performance observed on proline is expected, as it can be attributed to its unique cyclic structure, which cannot be generalized from any of the other amino acids.

It is crucial to note that both of these evaluations are very stringent as the trained model has never encountered the respective modification or amino acid on which it is being evaluated. Furthermore, for the second evaluation, peptides that are similar and thus likely to contain the excluded amino acid will be collectively omitted from training, adding to the challenge. This factor is especially pertinent for lysine and arginine because all peptides in these data sets are tryptic. The resulting bias in training sets could potentially impact the model’s generalization ability. Nevertheless, despite these challenges, our model demonstrates high accuracy in predicting CCS values for amino acids absent from its training data, indicating its robustness and flexibility.

#### Performance of the IM2Deep Model Shipped with TIMS^2^Rescore

**Figure 4:**
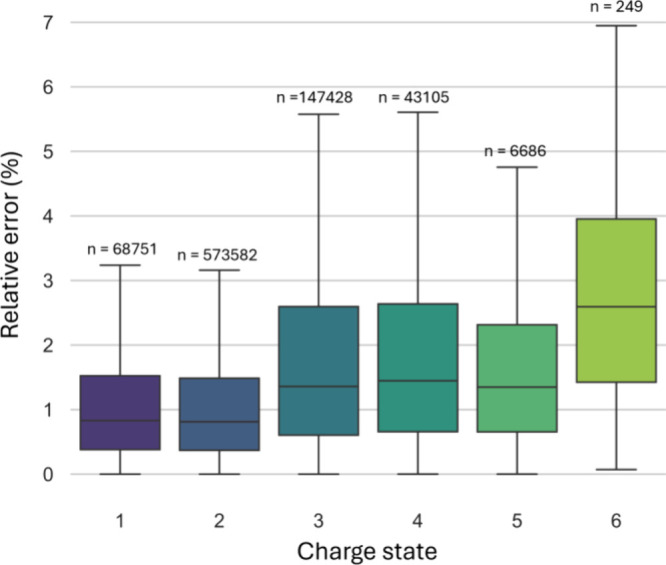
Box plots illustrating the relative error of the predictions made by the main IM2Deep model for peptides with different charge states. Numbers above the boxes indicate the number of training peptides with the corresponding charge state.

A combination of the aforementioned evaluation data set and an immunopeptide data set^20^ was used to train and evaluate the final IM2Deep model which is shipped with TIMS^2^Rescore. This data set was included to enhance IM2Deep’s performance on nontryptic peptides, and on peptides with charge states 1, 5, and 6. Evaluation on the test set (10%) shows a mean absolute error of 6.26 Å^2^, a median relative error of 0.91% and a PCC of 0.996 (Supplementary Figure S6). Good predictive performance is observed across all charge states present in the data set (Figure 4). The somewhat diminished performance on peptides with charge 6 can be explained by the limited number of training peptides (n = 249) for this charge state.

#### Rescoring Performance

**Figure 5:**
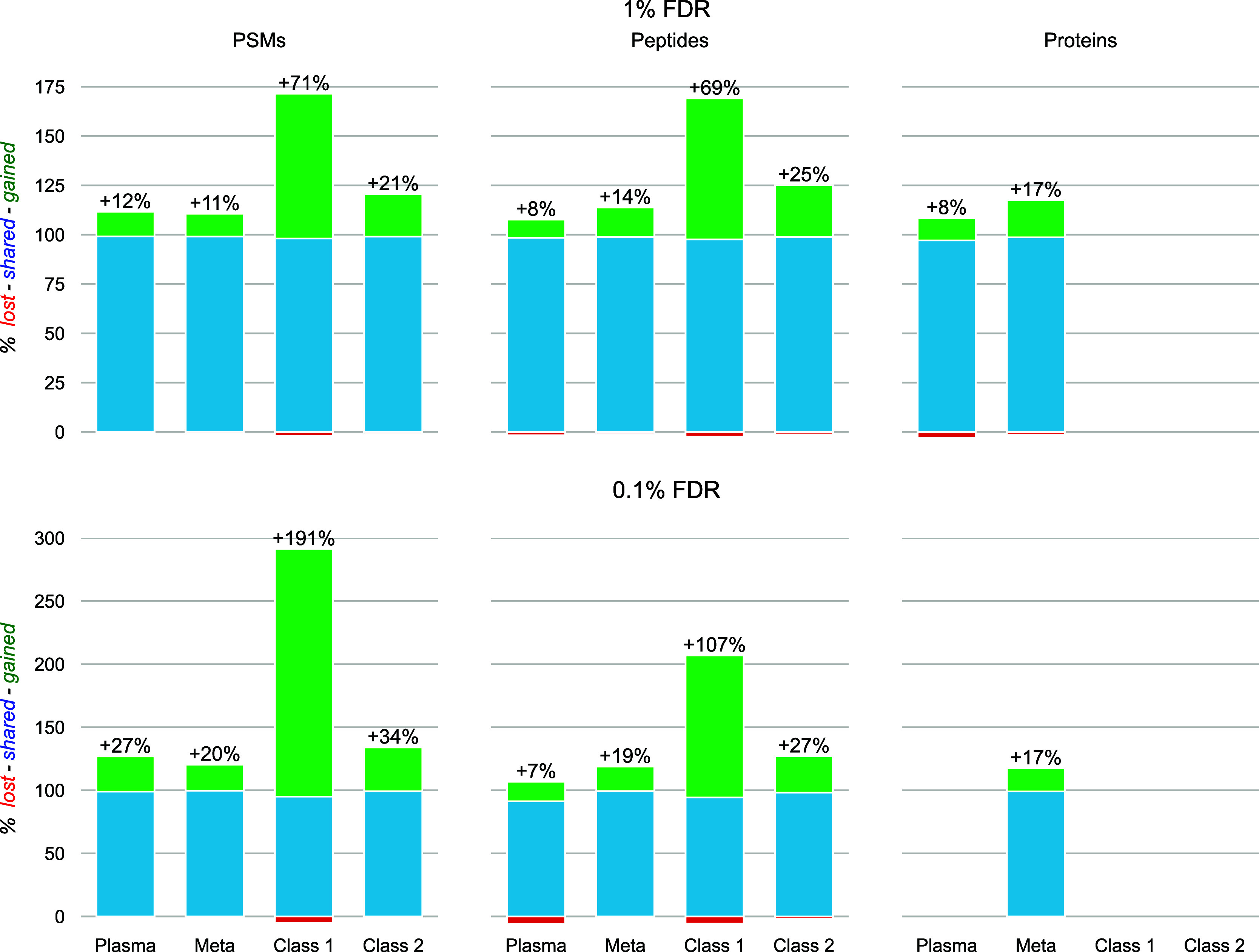
Gained (green), shared (blue), and lost (red) identifications at 1% and 0.1% PSM-, peptide-, and protein-level FDR, for Sage with TIMS^2^Rescore versus Sage without data-driven rescoring. Results for four data set types are shown: plasma proteomics (Plasma), gut metaproteomics (Meta), class I immunopeptides (Class I), and class II immunopeptides (Class II).

Data coming from various proteomics subfields were used to (i) assess the overall rescoring performance of TIMS^2^Rescore, and (ii) evaluate the performance of the newly trained prediction models. Overall, Figure 5 shows at least a 10% relative increase in confidently identified PSMs at 1% FDR and at least a 20% increase at 0.1% FDR compared to Sage without rescoring (see Supplementary Table S2 for identification counts). Most notably, we see a very large increase of almost 71% (191% at 0.1% FDR) for the class I immunopeptides, which could be partially explained by the very large amount of data used for simultaneous rescoring, compared to the other data sets, leading to a robust rescoring model. Indeed, while Sage already identified 758,096 PSMs prior to rescoring for class I immunopeptides, it only identified 83,412, 177,862 and 214,261 PSMs for plasma, meta proteomics, and class II immunopeptides, respectively. Similar gains on the peptide level are also seen for all data types, where only the plasma proteomics data set has a slightly lower increase in peptide identifications than in PSMs. This is due to the few highly abundant proteins generating repeated spectrum identifications for the same peptides, such as albumin. Nevertheless, the newly identified peptides still lead to an 8% increase in plasma protein identifications. Similarly, for a highly complex sample such as gut metaproteomics, we observe a substantial increase in protein identifications. Moreover, the rescoring features from the new prediction models (PCC for the MS^2^PIP model and CCS error for IM2Deep) consistently show high correlations and low absolute errors, respectively, for confidently identified PSMs (Supplementary Figure S7-8). The rescoring algorithm assigns high weights to the MS^2^PIP features, emphasizing the importance of the new MS^2^PIP models. However, despite their low errors, the IM2Deep features receive lower weights, indicating that these features have a lower impact on rescoring (Supplementary Figure S9). Most likely, this is due to the low orthogonality of CCS values with other features such as *m*/*z* and charge, as was previously described.^31^ Nevertheless, these features could still prove useful to further boost discrimination between similar candidate PSMs. This is further corroborated by running each feature generator separately for the plasma data, where we see a similar effect (Supplementary Figure S10).

Despite the new prediction models being primarily trained and evaluated on human data, no bias toward human-derived samples was observed. An entrapment experiment, where *E. coli* was analyzed using *H. sapiens* and *P. furiosus* databases as entrapments, respectively, demonstrated comparable percentages of false hits before and after rescoring (Supplementary Figure S11). While human entrapments did consistently yield higher percentages, this can be attributed to the potential presence of human proteins in the samples, either due to contamination or due to carry-over. This is supported by comparing the PCC of the predicted versus observed spectra for PSMs associated with the most abundant human protein, which were significantly higher than those for decoys, suggesting the genuine presence of these peptides in the sample (Supplementary Figure S11). Overall, we present a robust rescoring method tailored for DDA-PASEF data, enhanced by new, high-performing prediction models.

## Discussion

Data-driven PSM rescoring is becoming a part of routine data analysis pipelines and has been repeatedly shown to greatly improve identification sensitivity and specificity.^12−14^ Simultaneously, state-of-the-art mass spectrometers, such as timsTOF instruments, provide increasingly higher sensitivities, pushing forward challenging proteomics subfields such as immunopeptidomics, plasma proteomics, and metaproteomics. Combining both technologies results in a much more performant analysis of a sample’s proteome.

We present a data-driven rescoring workflow optimized for timsTOF instruments. It includes new MS^2^PIP spectrum prediction models that accurately predict fragmentation behavior in timsTOF instruments for a wide range of peptide types. While the new timsTOF MS^2^PIP models reach somewhat lower accuracies than what would be expected for Orbitrap data, correlations between observed timsTOF spectra indicate that this is a limiting factor inherent to the data, rather than from the prediction models. Moreover, the predictions do provide the expected boost in identification rates when used for PSM rescoring. This is reflected in the weights of the MS^2^PIP-derived features in the rescoring model. Furthermore, we included CCS predictions to further remove ambiguity for harder to identify PSMs, as was previously done for singly charged immunopeptides.^31^ With TIMS^2^Rescore, we provide a straightforward interface to the specialized timsTOF models for data-driven rescoring with direct support for Bruker raw spectrum files, thus alleviating the need for often slow and cumbersome file conversion steps. Together with the support for ProteoScape PSM files, as well as many other search engine PSM files, these new features make TIMS^2^Rescore an ideal postprocessing tool for timsTOF identification workflows.

We showcased the performance of TIMS^2^Rescore on several publicly available data sets of class I and class II immunopeptides, plasma proteomics, and metaproteomics experiments. Overall, TIMS^2^Rescore lead to gains of at least 10% in confidently identified PSMs, with more drastic gains for the larger and more challenging data sets, such as the class I immunopeptides. The plasma proteomics and metaproteomics data sets show similar gains at the protein level with 8% and 17%, respectively.

## Conclusions

Recent advancements in proteomics, such as highly sensitive mass spectrometers and the integration of AI, are clearly pushing the field forward. These technologies have made it possible to identify low abundant proteins with higher specificity, enhancing our understanding of biology and disease mechanisms. The development and application of tools like TIMS^2^Rescore with the newly trained MS^2^PIP and IM2Deep models demonstrate the positive impact of combining cutting-edge instrumentation with computational innovations. As we continue to leverage these technological advancements, the potential for new discoveries and improvements in disease diagnosis and treatment is vast.

## Data Availability

TIMS^2^Rescore is freely available and open source under the permissive Apache-2.0 license. It is available as a specialized command in the MS^2^Rescore Python package and is distributed through PyPI, Bioconda, and Biocontainers. The source code is available on GitHub at https://github.com/compomics/tims2rescore. IM2Deep is open source under the permissive Apache-2.0 license and is freely available within TIMS^2^Rescore and as a stand-alone Python package on PyPI. The source code is available on GitHub at https://github.com/compomics/IM2Deep. All data and scripts required to reproduce the presented results is available on Zenodo at 10.5281/zenodo.11277943.

## Supporting Information Available

Generations of the plasma proteomics and class II immunopeptidomics samples; (Table S1) median Pearson correlation coefficients between predicted and observed intensities for different MS^2^PIP models; (Table S2) absolute numbers of identifications lost, shared, and gained for each rescored data set; (Figure S1) variability between different spectra from the same peptidoform; (Figure S2) relation between observed and predicted CCS values for peptides with a certain modification, for which the model was not trained; (Figure S3) relation between the Tanimoto similarity of the most similar modification and the performance improvement when unseen modifications are encoded compared to when they are ignored; (Figure S4) relation between training set sizes of different IM2Deep models not trained on specific modifications and the performance improvement when unseen modifications are encoded compared to when they are ignored; (Figure S5) improvement in CCS prediction accuracy for peptides with modifications and amino acids unseen during training when these are encoded during testing compared to when they are ignored; (Figure S6) overall performance of the IM2Deep model within MS^2^Rescore; (Figure S7) percentual CCS errors for rescoring; (Figure S8) Pearson correlation coefficients for rescoring; (Figure S9) feature weights for rescoring class I immunopeptides, color coded by feature generator; (Figure S10) individual performances of the feature generators for the plasma data set; (Figure S11) entrapment analysis using TIMS^2^Recore (PDF)

## References

[ref1] Al-Amrani S.; Al-Jabri Z.; Al-Zaabi A.; Alshekaili J.; Al-Khabori M. Proteomics: Concepts and Applications in Human Medicine. World J. Biol. Chem. 2021, 12 (5), 57 10.4331/wjbc.v12.i5.57. 34630910 PMC8473418

[ref2] Moseley F. L.; Bicknell K. A.; Marber M. S.; Brooks G. The Use of Proteomics to Identify Novel Therapeutic Targets for the Treatment of Disease. J. Pharm. Pharmacol. 2007, 59 (5), 609–628. 10.1211/jpp.59.5.0001. 17524226

[ref3] Kleiner M. Metaproteomics: Much More than Measuring Gene Expression in Microbial Communities. mSystems 2019, 4 (3), e00115-19 10.1128/MSYSTEMS.00115-19. 31117019 PMC6529545

[ref4] Van Den Bossche T.; Kunath B. J.; Schallert K.; Schäpe S. S.; Abraham P. E.; Armengaud J.; Arntzen M.; Bassignani A.; Benndorf D.; Fuchs S.; Giannone R. J.; Griffin T. J.; Hagen L. H.; Halder R.; Henry C.; Hettich R. L.; Heyer R.; Jagtap P.; Jehmlich N.; Jensen M.; Juste C.; Kleiner M.; Langella O.; Lehmann T.; Leith E.; May P.; Mesuere B.; Miotello G.; Peters S. L.; Pible O.; Queiros P. T.; Reichl U.; Renard B. Y.; Schiebenhoefer H.; Sczyrba A.; Tanca A.; Trappe K.; Trezzi J. P.; Uzzau S.; Verschaffelt P.; von Bergen M.; Wilmes P.; Wolf M.; Martens L.; Muth T. Critical Assessment of MetaProteome Investigation (CAMPI): A Multi-Laboratory Comparison of Established Workflows. Nat. Commun. 2021, 12 (1), 1–15. 10.1038/s41467-021-27542-8. 34911965 PMC8674281

[ref5] Ignjatovic V.; Geyer P. E.; Palaniappan K. K.; Chaaban J. E.; Omenn G. S.; Baker M. S.; Deutsch E. W.; Schwenk J. M. Mass Spectrometry-Based Plasma Proteomics: Considerations from Sample Collection to Achieving Translational Data. J. Proteome Res. 2019, 18 (12), 4085 10.1021/acs.jproteome.9b00503. 31573204 PMC6898750

[ref6] Phulphagar K. M.; Ctortecka C.; Jacome A. S. V.; Klaeger S.; Verzani E. K.; Hernandez G. M.; Udeshi N. D.; Clauser K. R.; Abelin J. G.; Carr S. A. Sensitive, High-Throughput HLA-I and HLA-II Immunopeptidomics Using Parallel Accumulation-Serial Fragmentation Mass Spectrometry. Mol. Cell. Proteom. 2023, 22 (6), 100563 10.1016/j.mcpro.2023.100563. PMC10326702 37142057

[ref7] Colaert N.; Degroeve S.; Helsens K.; Martens L. Analysis of the Resolution Limitations of Peptide Identification Algorithms. J. Proteome Res. 2011, 10 (12), 5555–5561. 10.1021/pr200913a. 21995378

[ref8] Bouwmeester R.; Gabriels R.; Van Den Bossche T.; Martens L.; Degroeve S. The Age of Data-Driven Proteomics: How Machine Learning Enables Novel Workflows. Proteomics 2020, 20 (21–22), 1900351 10.1002/pmic.201900351. 32267083

[ref9] Neely B. A.; Dorfer V.; Martens L.; Bludau I.; Bouwmeester R.; Degroeve S.; Deutsch E. W.; Gessulat S.; Käll L.; Palczynski P.; Payne S. H.; Rehfeldt T. G.; Schmidt T.; Schwämmle V.; Uszkoreit J.; Vizcaíno J. A.; Wilhelm M.; Palmblad M. Toward an Integrated Machine Learning Model of a Proteomics Experiment. J. Proteome Res. 2023, 22 (3), 681–696. 10.1021/acs.jproteome.2c00711. 36744821 PMC9990124

[ref10] Bouwmeester R.; Gabriels R.; Hulstaert N.; Martens L.; Degroeve S. DeepLC Can Predict Retention Times for Peptides That Carry As-yet Unseen Modifications. Nat. Methods 2021, 1–7. 10.1038/s41592-021-01301-5. 34711972

[ref11] Degroeve S.; Martens L. MS2PIP: A Tool for MS/MS Peak Intensity Prediction. Bioinformatics 2013, 29 (24), 3199–3203. 10.1093/bioinformatics/btt544. 24078703 PMC5994937

[ref12] Buur L. M.; Declercq A.; Strobl M.; Bouwmeester R.; Degroeve S.; Martens L.; Dorfer V.; Gabriels R. MS2Rescore 3.0 Is a Modular, Flexible, and User-Friendly Platform to Boost Peptide Identifications, as Showcased with MS Amanda 3.0. J. Proteome Res. 2023, 3200 10.1021/ACS.JPROTEOME.3C00785. 38491990

[ref13] Picciani M.; Gabriel W.; Giurcoiu V. G.; Shouman O.; Hamood F.; Lautenbacher L.; Jensen C. B.; Müller J.; Kalhor M.; Soleymaniniya A.; Kuster B.; The M.; Wilhelm M. Oktoberfest: Open-Source Spectral Library Generation and Rescoring Pipeline Based on Prosit. Proteomics 2024, 24 (8), 2300112 10.1002/pmic.202300112. 37672792

[ref14] C. Silva A. S.; Bouwmeester R.; Martens L.; Degroeve S.; Wren J. Accurate Peptide Fragmentation Predictions Allow Data Driven Approaches to Replace and Improve upon Proteomics Search Engine Scoring Functions. Bioinformatics 2019, 35 (24), 5243–5248. 10.1093/bioinformatics/btz383. 31077310

[ref15] Declercq A.; Bouwmeester R.; Hirschler A.; Carapito C.; Degroeve S.; Martens L.; Gabriels R. MS2Rescore: Data-Driven Rescoring Dramatically Boosts Immunopeptide Identification Rates. Mol. Cell. Proteomics 2022, 21 (8), 100266 10.1016/j.mcpro.2022.100266. 35803561 PMC9411678

[ref16] Wilhelm M.; Zolg D. P.; Graber M.; Gessulat S.; Schmidt T.; Schnatbaum K.; Schwencke-Westphal C.; Seifert P.; de Andrade Krätzig N.; Zerweck J.; Knaute T.; Bräunlein E.; Samaras P.; Lautenbacher L.; Klaeger S.; Wenschuh H.; Rad R.; Delanghe B.; Huhmer A.; Carr S. A.; Clauser K. R.; Krackhardt A. M.; Reimer U.; Kuster B. Deep Learning Boosts Sensitivity of Mass Spectrometry-Based Immunopeptidomics. Nat. Commun. 2021, 12 (1), 1–12. 10.1038/s41467-021-24263-w. 34099720 PMC8184761

[ref17] Li K.; Jain A.; Malovannaya A.; Wen B.; Zhang B. DeepRescore: Leveraging Deep Learning to Improve Peptide Identification in Immunopeptidomics. Proteomics 2020, 20, 21–22. 10.1002/pmic.201900334. PMC7718998 32864883

[ref18] Meier F.; Beck S.; Grassl N.; Lubeck M.; Park M. A.; Raether O.; Mann M. Parallel Accumulation-Serial Fragmentation (PASEF): Multiplying Sequencing Speed and Sensitivity by Synchronized Scans in a Trapped Ion Mobility Device. J. Proteome Res. 2015, 14 (12), 5378–5387. 10.1021/acs.jproteome.5b00932. 26538118

[ref19] Gomez-Zepeda D.; Arnold-Schild D.; Beyrle J.; Declercq A.; Gabriels R.; Kumm E.; Preikschat A.; Łącki M. K.; Hirschler A.; Rijal J. B.; Carapito C.; Martens L.; Distler U.; Schild H.; Tenzer S. Thunder-DDA-PASEF Enables High-Coverage Immunopeptidomics and Is Boosted by MS2Rescore with MS2PIP TimsTOF Fragmentation Prediction Model. Nat. Commun. 2024, 15 (1), 1–18. 10.1038/s41467-024-46380-y. 38480730 PMC10937930

[ref20] Hoenisch Gravel N.; Nelde A.; Bauer J.; Mühlenbruch L.; Schroeder S. M.; Neidert M. C.; Scheid J.; Lemke S.; Dubbelaar M. L.; Wacker M.; Dengler A.; Klein R.; Mauz P. S.; Löwenheim H.; Hauri-Hohl M.; Martin R.; Hennenlotter J.; Stenzl A.; Heitmann J. S.; Salih H. R.; Rammensee H. G.; Walz J. S. TOFIMS Mass Spectrometry-Based Immunopeptidomics Refines Tumor Antigen Identification. Nat. Commun. 2023, 14 (1), 1–12. 10.1038/s41467-023-42692-7. 37978195 PMC10656517

[ref21] Vitko D.; Chou W. F.; Nouri Golmaei S.; Lee J. Y.; Belthangady C.; Blume J.; Chan J. K.; Flores-Campuzano G.; Hu Y.; Liu M.; Marispini M. A.; Mora M. G.; Ramaswamy S.; Ranjan P.; Williams P. B.; Zawada R. J. X.; Ma P.; Wilcox B. E. TimsTOF HT Improves Protein Identification and Quantitative Reproducibility for Deep Unbiased Plasma Protein Biomarker Discovery. J. Proteome Res. 2024, 23, 929–938. 10.1021/acs.jproteome.3c00646. 38225219 PMC10913052

[ref22] Chen T.; Guestrin C. XGBoost: A Scalable Tree Boosting System. In Proceedings of the 22nd ACM SIGKDD International Conference on Knowledge Discovery and Data Mining; ACM: New York, NY, USA. 10.1145/2939672.

[ref23] Meier F.; Köhler N. D.; Brunner A. D.; Wanka J. M. H.; Voytik E.; Strauss M. T.; Theis F. J.; Mann M. Deep Learning the Collisional Cross Sections of the Peptide Universe from a Million Experimental Values. Nat. Commun. 2021, 12 (1), 1–12. 10.1038/s41467-021-21352-8. 33608539 PMC7896072

[ref24] Will A.; Oliinyk D.; Bleiholder C.; Meier F. Peptide Collision Cross Sections of 22 Post-Translational Modifications. Anal Bioanal Chem. 2023, 415 (27), 6633–6645. 10.1007/s00216-023-04957-4. 37758903 PMC10598134

[ref25] Prianichnikov N.; Koch H.; Koch S.; Lubeck M.; Heilig R.; Brehmer S.; Fischer R.; Cox J. MaxQuant Software for Ion Mobility Enhanced Shotgun Proteomics. Molecular & Cellular Proteomics 2020, 19 (6), 1058–1069. 10.1074/mcp.TIR119.001720. 32156793 PMC7261821

[ref26] Lazear M. R. Sage: An Open-Source Tool for Fast Proteomics Searching and Quantification at Scale. J. Proteome Res. 2023, 22 (11), 3652–3659. 10.1021/acs.jproteome.3c00486. 37819886

[ref27] Van Puyvelde B.; Daled S.; Willems S.; Gabriels R.; Gonzalez de Peredo A.; Chaoui K.; Mouton-Barbosa E.; Bouyssié D.; Boonen K.; Hughes C. J.; Gethings L. A.; Perez-Riverol Y.; Bloomfield N.; Tate S.; Schiltz O.; Martens L.; Deforce D.; Dhaenens M. A Comprehensive LFQ Benchmark Dataset on Modern Day Acquisition Strategies in Proteomics. Scientific Data 2022, 9 (1), 1–12. 10.1038/s41597-022-01216-6. 35354825 PMC8967878

[ref28] Frankenfield A. M.; Ni J.; Ahmed M.; Hao L. Protein Contaminants Matter: Building Universal Protein Contaminant Libraries for DDA and DIA Proteomics. J. Proteome Res. 2022, 21 (9), 2104–2113. 10.1021/acs.jproteome.2c00145. 35793413 PMC10040255

[ref29] Gabriels R.; Martens L.; Degroeve S. Updated MS^2^PIP Web Server Delivers Fast and Accurate MS^2^ Peak Intensity Prediction for Multiple Fragmentation Methods, Instruments and Labeling Techniques. Nucleic Acids Res. 2019, 47 (W1), W295–W299. 10.1093/nar/gkz299. 31028400 PMC6602496

[ref30] Adams C.; Gabriel W.; Laukens K.; Picciani M.; Wilhelm M.; Bittremieux W.; Boonen K. Fragment Ion Intensity Prediction Improves the Identification Rate of Non-Tryptic Peptides in TimsTOF. Nat. Commun. 2024, 15 (1), 1–11. 10.1038/s41467-024-48322-0. 38730277 PMC11087512

[ref31] Teschner D.; Gomez-Zepeda D.; Declercq A.; Łącki M. K.; Avci S.; Bob K.; Distler U.; Michna T.; Martens L.; Tenzer S.; Hildebrandt A. Ionmob: A Python Package for Prediction of Peptide Collisional Cross-Section Values. Bioinformatics 2023, 39 (9), btad486 10.1093/BIOINFORMATICS/BTAD486. 37540201 PMC10521631

