# Supplementary material for: TIMS2Rescore: A Data Dependent Acquisition-Parallel Accumulation and Serial Fragmentation-Optimized Data-Driven Rescoring Pipeline Based on MS2Rescore
Source: J Proteome Res. 2025 Feb 7;24(3):1067–76. doi: 10.1021/acs.jproteome.4c00609 (PMC11894666; doi:10.1021/acs.jproteome.4c00609)
Supplement: Supplementary file 1 — pr4c00609_si_001.pdf [file pr4c00609_si_001.pdf]

TIMS²Rescore: A DDA-PASEF optimized data-driven rescoring pipeline based on MS²Rescore

Arthur Declercq[1, 2, §], Robbe Devreese[1, 2, §], Jonas Scheid[3, 4, 5], Caroline Jachmann[1, 2], Tim Van Den Bossche[1, 2], Annica Preikschat[6], David Gomez-Zepeda[7, 8], Jeewan Babu Rijal[9], Aurélie Hirschler[9], Jonathan R Krieger[10], Tharan Srikumar[10], George Rosenberger[11], Claudia Martelli[11], Dennis Trede[12], Christine Carapito[9], Stefan Tenzer[6, 7, 13], Juliane S Walz[3, 4, 14, 15], Sven Degroeve[1, 2], Robbin Bouwmeester[1, 2], Lennart Martens*[1, 2, 9], Ralf Gabriels[1, 2]

[§] contributed equally
[*] corresponding author

[1] VIB-UGent Center for Medical Biotechnology, VIB, Ghent 9052 Belgium
[2] Department of Biomolecular Medicine, Ghent University, Ghent 9052 Belgium
[3] Department of Peptide-based Immunotherapy, Institute of Immunology, University and University Hospital Tübingen, Tübingen 72076, Germany
[4] Cluster of Excellence iFIT (ECX2180) Image-Guided and Functionally Instructed Tumor Therapies, University of Tuebingen, Tuebingen 72076, Germany
[5] Quantitative Biology Center (QBiC), University of Tübingen, Tübingen 72076, Germany
[6] Institute of Immunology, University Medical Center of the Johannes-Gutenberg University, Mainz 55131, Germany
[7] Helmholtz Institute for Translational Oncology Mainz (HI-TRON Mainz) – A Helmholtz Institute of the DKFZ, Mainz 55131, Germany
[8] German Cancer Research Center (DKFZ) Heidelberg, Division 191 & Immunopeptidomics Platform, Heidelberg 69120, Germany
[9] BioOrganic Mass Spectrometry Laboratory (LSMBO), IPHC UMR 7178, University of Strasbourg, CNRS, ProFI FR2048, Strasbourg 67087, France
[10] Bruker Ltd., Milton, Ontario L9T 6P4, Canada
[11] Bruker Switzerland AG, Faellanden 8117, Switzerland
[12] Bruker Daltonics GmbH & Co. KG, Bremen 28359, Germany
[13] Research Center for Immunotherapy (FZI), University Medical Center of the Johannes-Gutenberg University, Mainz 55131, Germany
[14] Clinical Collaboration Unit Translational Immunology, Department of Internal Medicine, University Hospital Tuebingen, Tuebingen 72076, Germany
[15] German Cancer Consortium (DKTK) and German Cancer Research Center (DKFZ), partner site Tübingen, Tübingen, 72076 Germany.

Table of content

Supplementary methods	Supplementary information on the generations of the plasma proteomics and class II immunopeptidomics samples.
Supplementary Table S1	Table showing the median Pearson correlation coefficients between predicted and observed intensities for different MS^2PIP models.
Supplementary Table S2	Table showing the absolute numbers of identifications lost, shared, and gained for each rescored data set.
Supplementary Figure S1	Figure showcasing the variability between different spectra from the same peptidoform.
Supplementary Figure S2	Figure showcasing relation between observed and predicted CCS values for peptides with a certain modification, for which the model was not trained.
Supplementary Figure S3	Figure showcasing relation between the Tanimoto similarity of the most similar modification and the performance improvement when unseen modifications are encoded compared to when they are ignored.
Supplementary Figure S4	Figure showcasing relation between training set sizes of different IM2Deep models not trained on specific modifications and the performance improvement when unseen modifications are encoded compared to when they are ignored.
Supplementary Figure S5	Figure showcasing the improvement in CCS prediction accuracy for peptides with modifications and amino acids unseen during training when these are encoded during testing compared to when they are ignored.
Supplementary Figure S6	Figure showcasing the overall performance of the IM2Deep model within $MS^2Rescore$.
Supplementary Figure S7	Figure showing percentual CCS errors for rescoring.
Supplementary Figure S8	Figure showing Pearson correlation coefficients for rescoring.
Supplementary Figure S9	Figure showing the feature weights for rescoring class I immunopeptides, color coded by feature generator.
Supplementary Figure S10	Figure showing the individual performance of the feature generators for the plasma dataset
Supplementary Figure S11	Figure showing the entrapment analysis

Supplementary Methods

Immunopeptide class II data set generation

Cell culture and harvesting

The human B lymphoblastoid cell line JY (CVCL_0108) was purchased from ATCC. Cells were maintained in RPMI1640 medium supplemented with 10 % FCS (Gibco (v/v)), 1 mM sodium pyruvate, 100 units/ml penicillin, and 100 µg/ml streptomycin. Cells were harvested at 220 x g for 10 min, washed three times with 1x PBS prior to counting, and frozen at -80°C until further use.

Immuno-affinity purification of HLA peptide ligands

HLA class II ligands were enriched by immunoprecipitation as described by (30) with modifications (31). Briefly, the cell pellets were thawed and lysed in a non-denaturant buffer (1% CHAPS in PBS (m/v)) aided by sonication. Immunoprecipitation was performed using the anti-HLA-DR antibody L243 immobilized on CNBr-activated beads (Cytivia). The monoclonal antibody was purchased from Hoelzel-biotech and produced by Leinco Technologies (ref. H261). Samples were incubated overnight with the Antibody-beads, then washed once with PBS and once with water. Then, peptide ligands were eluted using 0.2% TFA (v/v) in water. Next, peptides were ultrafiltered using molecular weight cutoff (MWCO) filters (Vivacon 500, 10,000 MWCO Hydrosart, Sartorius). The flow-through was desalted by SPE on a Hydrophilic-Lipophilic-Balanced sorbent (Oasis HLB 96-well µElution Plate, 2 mg Sorbent per Well, 30 µm, Waters Corp.), applying 35% ACN (v/v), 0.1% TFA (v/v) for elution. The eluates were dried in a vacuum concentrator and dissolved in 15 µL of water with 0.1% FA (v/v) for subsequent LC-MS/MS analysis.

LC-MS analysis for HLA-DR immunopeptidomics profiling of JY cells

NanoLC-MS analysis was performed using a nanoElute coupled to a timsTOF Pro 2 mass spectrometer. The desalted peptides were directly injected in a C18 Reversed-phase (RP) Aurora 25 cm analytical column (25 cm x 75 µm ID, 120 Å pore size, 1.7 µm particle size, IonOpticks, Australia) and separated using a 47 min gradient increasing the proportion of phase B (ACN with 0.1% FA (v/v)) to phase A (water with 0.1% FA (v/v)). The gradient started at 2% B, which increased to 17% within 23 min, then to 25% in the next 11.5 min, to 37% in 3.8 min, and to 95% in 3.8 min, before a wash step of 4.9 min at 95% B. A Captive Spray source was used for ionization, with a capillary voltage of 1600 V, dry gas at 3.0 L/min, dry temperature at 180 °C, and TIMS-in pressure of 2.7 mBar. Data was acquired using Compass Hystar and timsControl (Bruker) in DDA-PASEF mode using settings based on (31). Ions were accumulated and resolved in 300 ms TIMS ramps from 0.65 to 1.75 Vs/cm^2, using three MS2 frames per cycle and a cycle overlap of one. A stepped isolation polygon including singly charged ions above 445 *m/z* was designed to select precursors with a positive charge from 1 to 5 for fragmentation. The fragmentation intensity threshold was set at 1000 and the target intensity at 20,000. The *m/z* acquisition range was set at 100 to 2000, and the high-sensitivity detection mode was activated.

Supplementary Tables

Supplementary Table S1: Table showing the median Pearson correlation coefficients between predicted and observed intensities for different MS^2PIP models and the median variance for multiple spectra from the same peptidoforms across different peptide types.

**Data type**	**Ion Type**	**Model**	**Prediction correlation**	**Peptidoform variance**
elastase	b	HCD	0,41	0,83
elastase	y	HCD	0,51	0,80
elastase	b	timsTOF2023	0,88	0,83
elastase	y	timsTOF2023	0,86	0,80
elastase	b	timsTOF2024	0,82	0,83
elastase	y	timsTOF2024	0,81	0,80
immuno class 1	b	HCD	0,37	0,84
immuno class 1	y	HCD	0,45	0,83
immuno class 1	b	timsTOF2023	0,90	0,84
immuno class 1	y	timsTOF2023	0,90	0,83
immuno class 1	b	timsTOF2024	0,88	0,84
immuno class 1	y	timsTOF2024	0,89	0,83
immuno class 2	b	HCD	0,63	0,76
immuno class 2	y	HCD	0,66	0,81
immuno class 2	b	timsTOF2023	0,63	0,76
immuno class 2	y	timsTOF2023	0,64	0,81
immuno class 2	b	timsTOF2024	0,83	0,76
immuno class 2	y	timsTOF2024	0,86	0,81
tryptic	b	HCD	0,41	0,75
tryptic	y	HCD	0,53	0,86
tryptic	b	timsTOF2023	0,86	0,75
tryptic	y	timsTOF2023	0,92	0,86
tryptic	b	timsTOF2024	0,85	0,75
tryptic	y	timsTOF2024	0,91	0,86

Supplementary Table S2: Tables showing the absolute numbers of lost, shared, and gained peptides when rescoring compared to no rescoring, where the upper table shows the count for a 1% FDR threshold and the lower table shows the 0.1% FDR threshold.

	**1% FDR**								
**Type**	**PSMs**			**Peptides**			**Proteins**		
**Status**	*lost*	*shared*	*gained*	*lost*	*shared*	*gained*	*lost*	*shared*	*gained*
**plasma**	724	82688	9649	70	4249	329	9	301	26
**metaproteomics**	1667	176195	18837	346	30226	4204	86	6548	1158
**class 1**	14825	743271	541540	1847	75358	53282	/	/	/
**class 2**	2109	211641	43934	226	18241	4633	/	/	/

	**0.1% FDR**								
**Type**	**PSMs**			**Peptides**			**Proteins**		
**Status**	*lost*	*shared*	*gained*	*lost*	*shared*	*gained*	*lost*	*shared*	*gained*
**plasma**	709	66477	18041	311	3289	243	/	/	/
**metaproteomics**	495	156725	32024	153	26928	5076	51	5829	1028
**class 1**	16532	314015	632238	2477	41187	46665	/	/	/
**class 2**	1379	173358	59391	286	15309	4201	/	/	/

Supplementary Figures

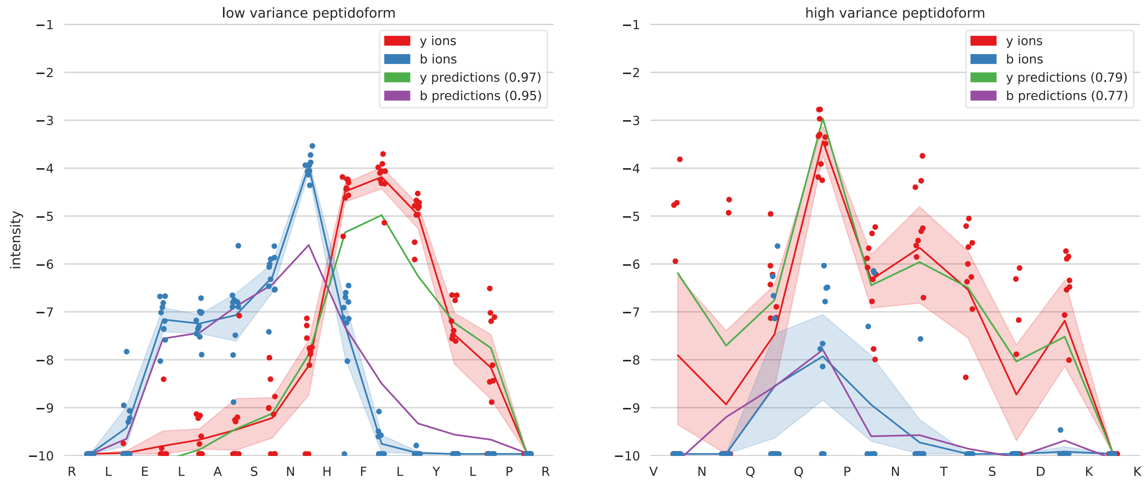

Supplementary Figure S1: line plot showing the intensity variation for two different peptides, one with a low variation and one with a high variation for b (blue) and y ions (red). The predictions of the 2024 timsTOF model are depicted in green for the y ions and purple for the b ions.

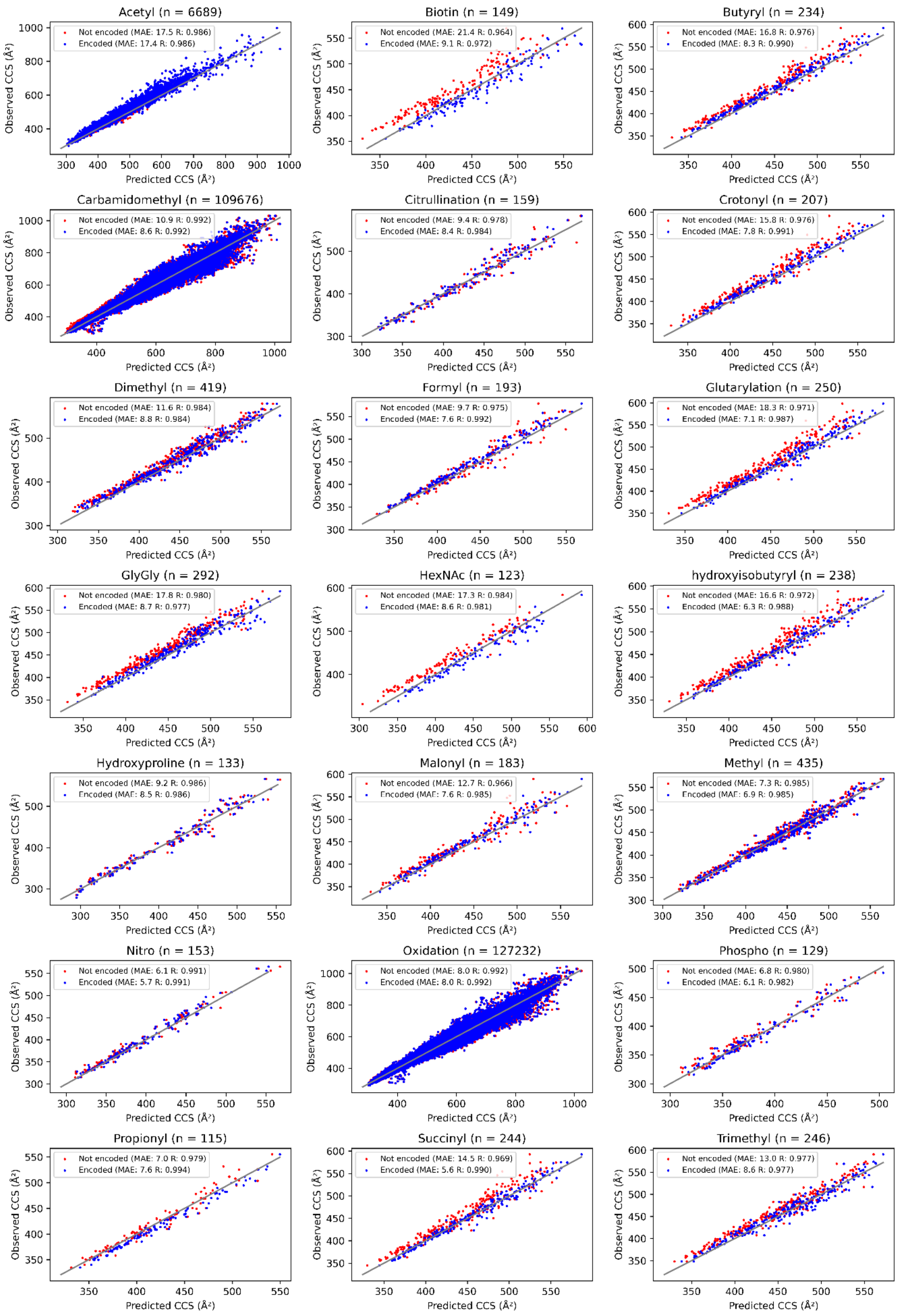

Supplementary Figure S2: Each scatter plot displays the observed CCS plotted against the CCS predicted by models that were not trained on peptides carrying the specified modification. The dots represent CCS when modifications were either not encoded (red) or encoded (blue) by IM2Deep. N denotes the total number of peptides with the specified modification.

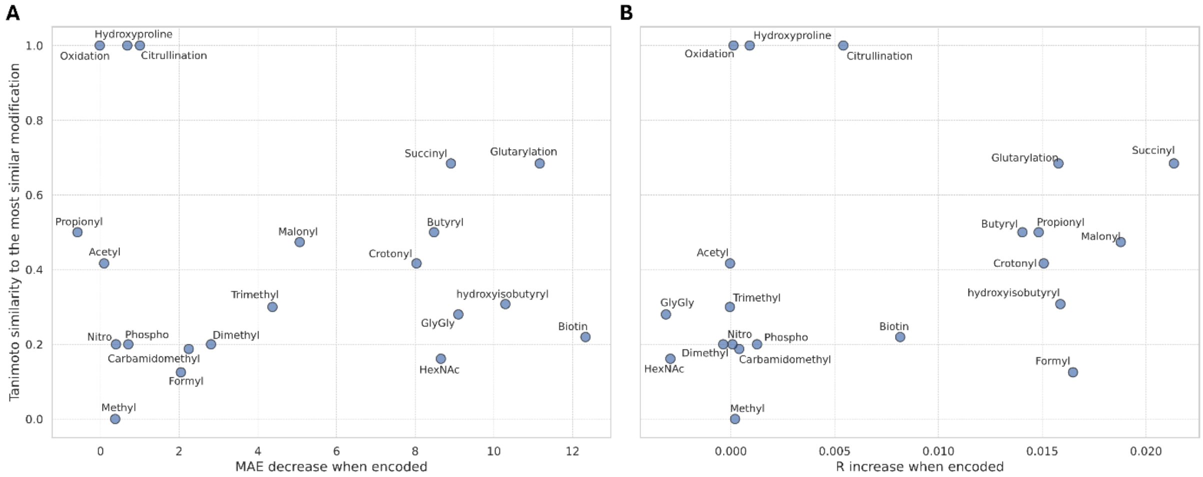

Supplementary Figure S3: Scatter plots displaying the MAE decrease (A) and Pearson R increase (B) when encoding unseen modifications for prediction compared to when they are ignored, against the Tanimoto similarity with the closest related modification.

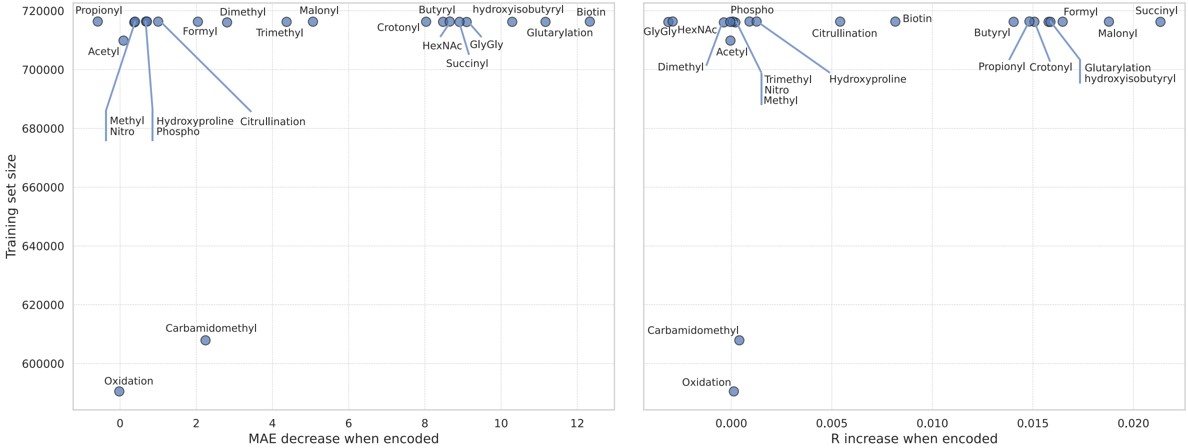

Supplementary Figure S4: Scatter plots displaying the MAE decrease (A) and Pearson R increase (B) when encoding unseen modifications for prediction compared to when they are ignored, against the training set size of the model that did not observe the specific modification.

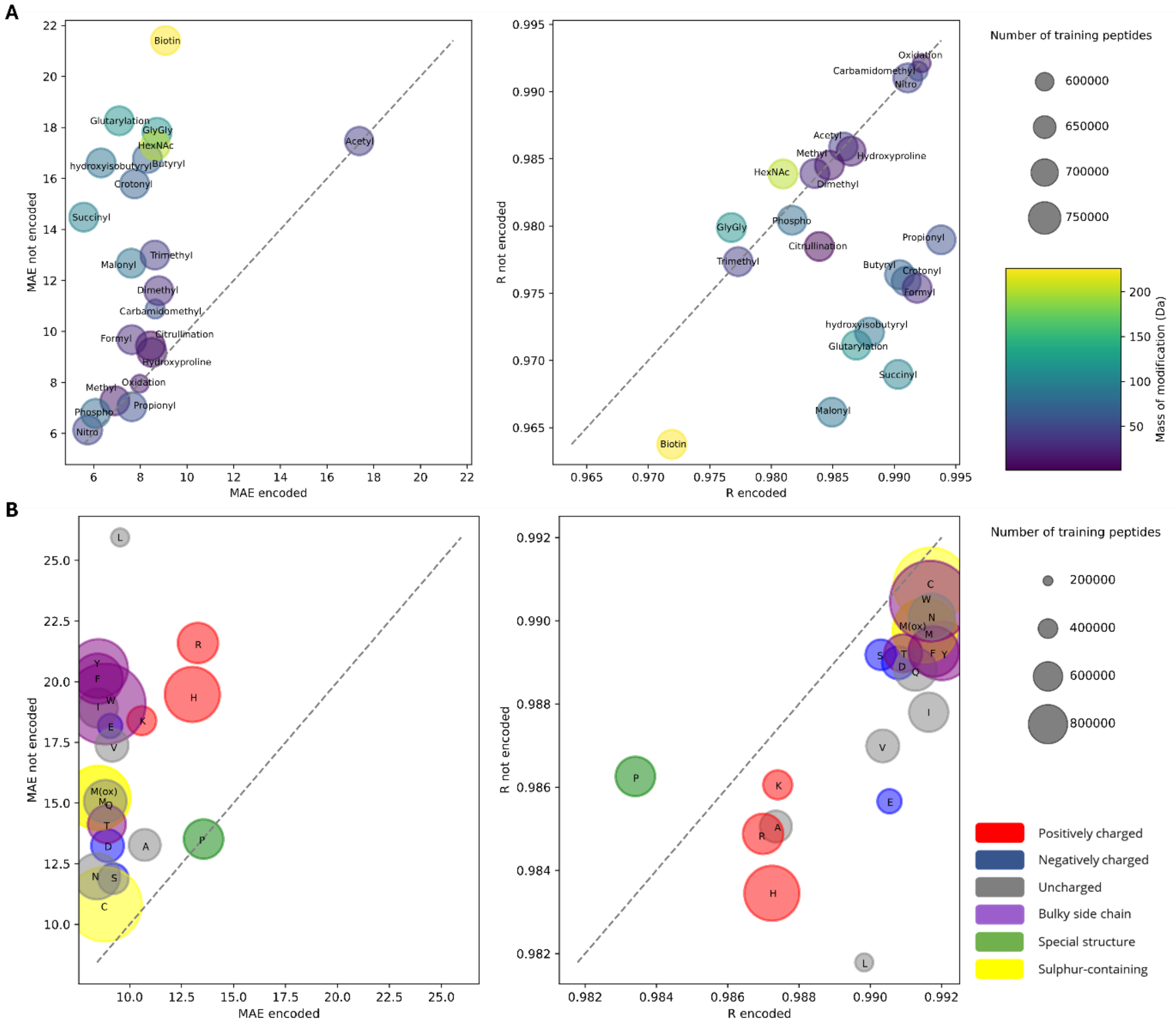

Supplementary Figure S5: Each modification (A) and amino acid (B) excluded during training is represented as a circle, where the circle's size indicates the remaining training peptides, and its color reflects the modification mass or the amino acid's chemical characteristic. (A) The modification is either not encoded (vertical axis) or encoded with its atomic composition (horizontal axis). (B) The amino acid is encoded either as glycine (vertical axis) or as its own atomic composition (horizontal axis). Circle positions indicate the MAE (left) or Pearson R (right) for all modification-carrying or amino acid-containing peptides.

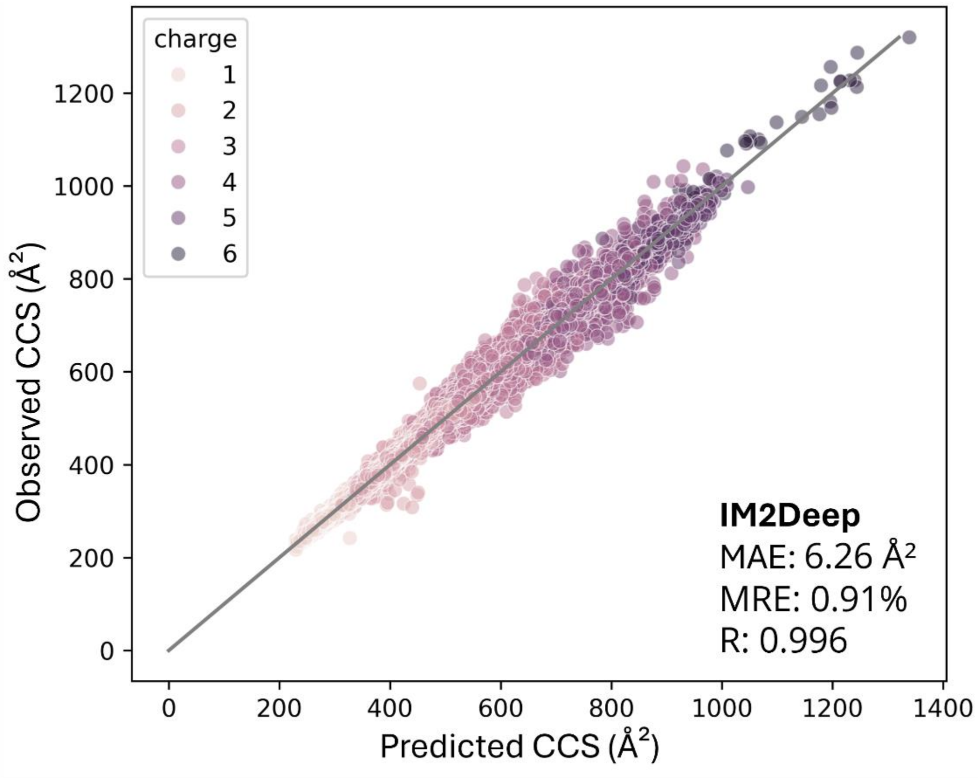

Supplementary Figure S6: Test set performance of the base IM2Deep model, shipped with TIMS²Rescore. MAE: mean absolute error, MRE: median relative error, R: Pearson correlation coefficient.

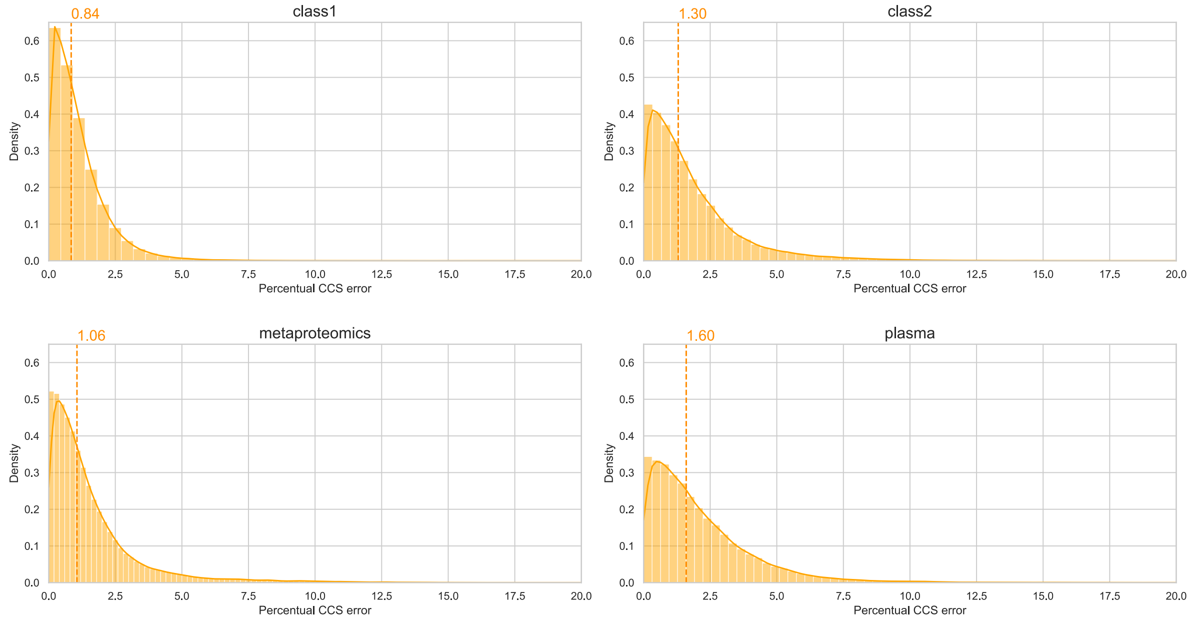

Supplementary Figure S7: KDE plots showing the distribution of percentual CCS error for all identified spectra after rescoring.

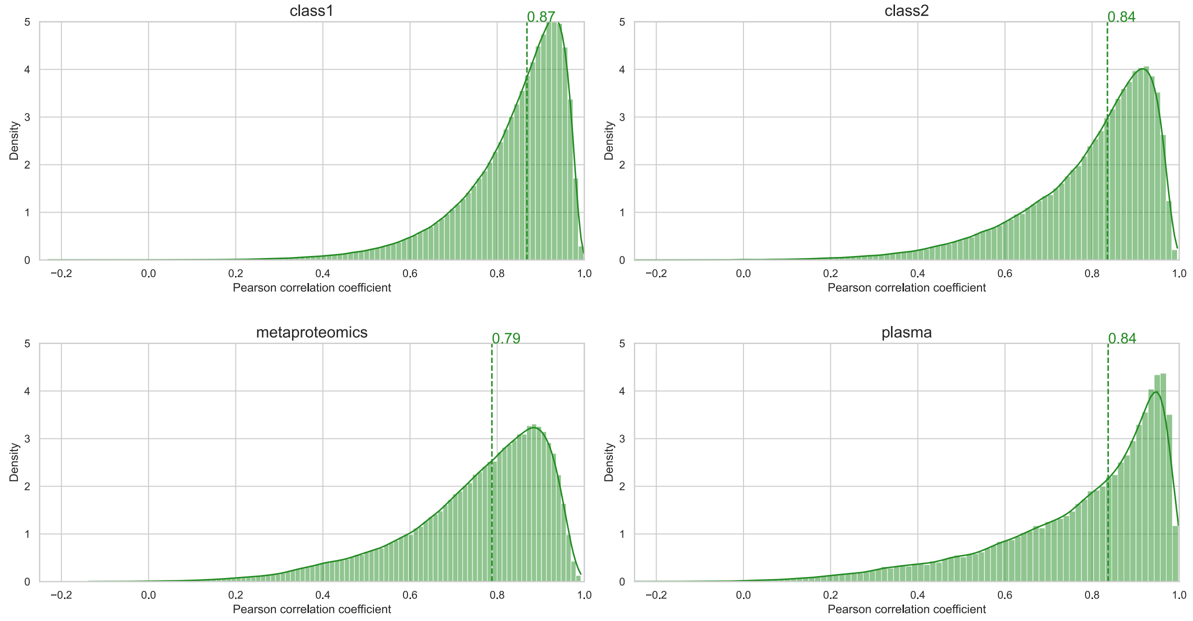

Supplementary Figure S8: Distributions of Pearson correlation coefficients for all identified spectra after rescoring.

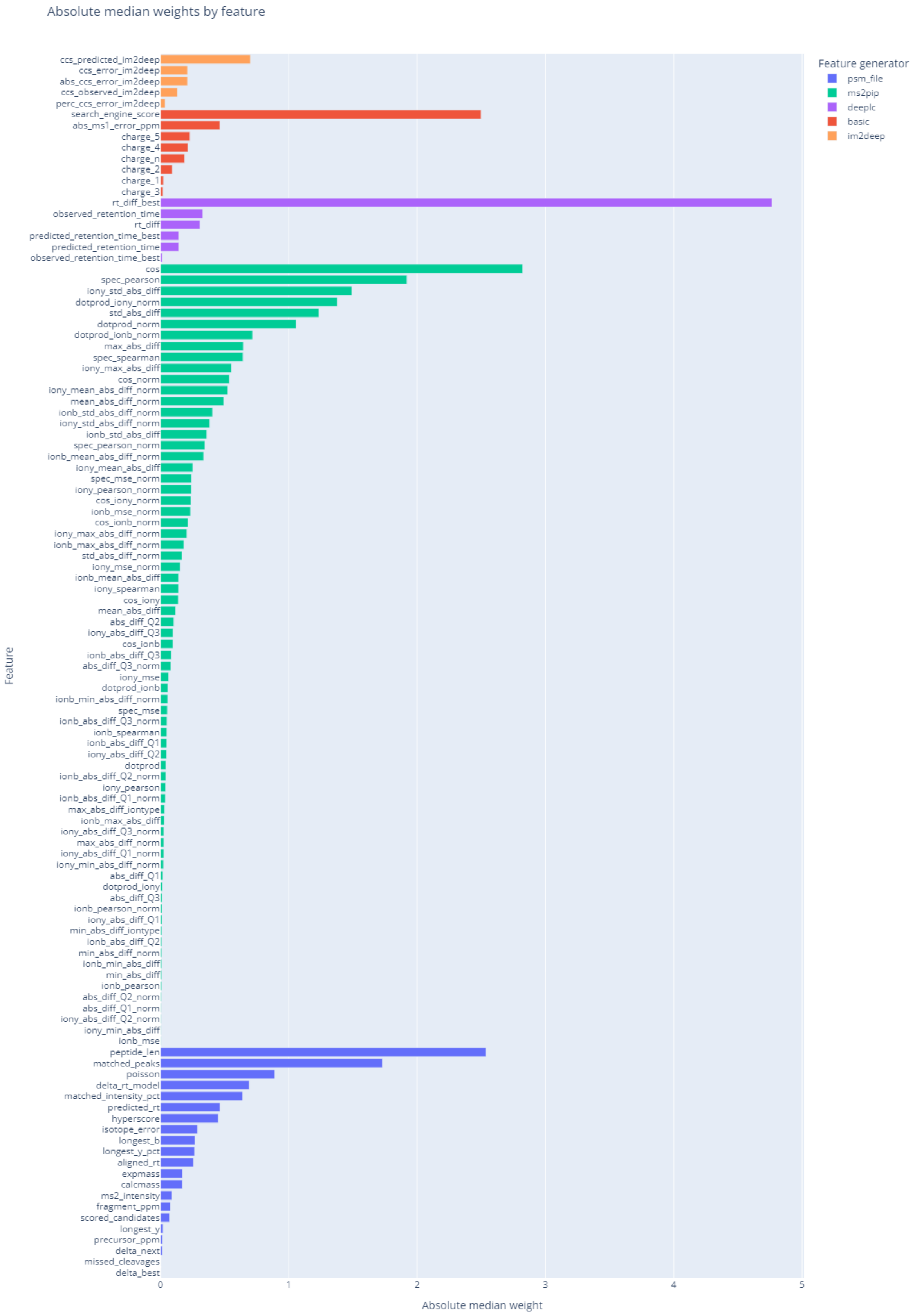

Supplementary Figure S9: Feature weights for the class I immunopeptides rescoring run color-coded for different feature generators.

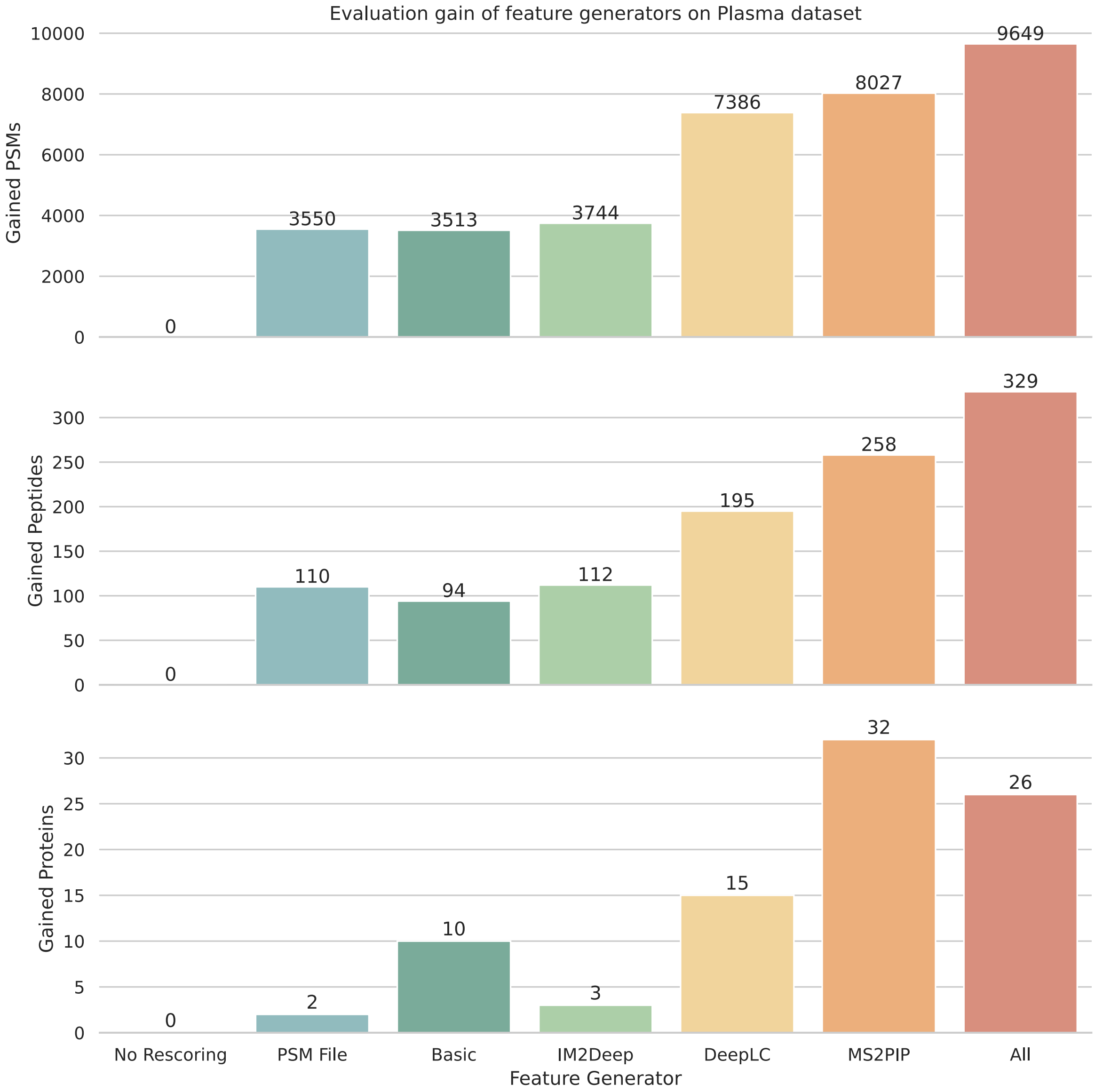

Supplementary Figure S10: Absolute gain in PSMs, peptides and proteins when each feature generator is used individually for rescoring, compared to no rescoring. For the rescoring runs with Basic, IM2Deep, DeepLC, and MS^{2}PIP feature generators, the feature sets were supplemented with the PSM file features, as these are search engine features that are typically used in any rescoring setting.

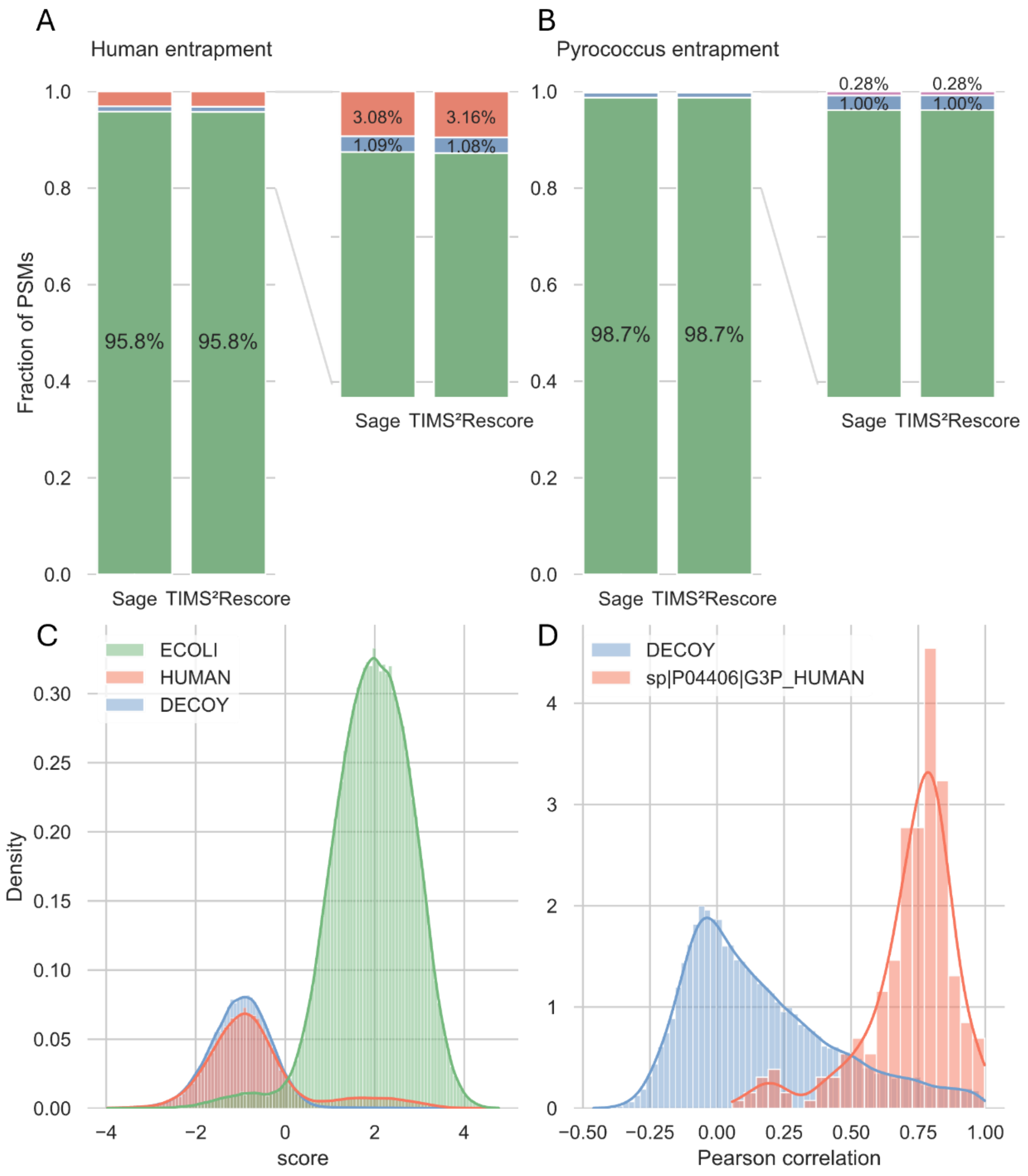

Supplementary Figure S11: Figure showing the entrapment percentages of both the H. sapiens *(A) and* P. furiosus *entrapment (B) experiment with* E. coli *(green),* H. sapiens / P. furiosus *(red) and decoys (blue) are shown. (C) Histogram showing the score distributions for the* H. sapiens *entrapment. (D) Histogram showing the Pearson correlation coefficients between observed and predicted spectra for PSMs of the SP|P04406|G3P_HUMAN protein, compared to the decoy distribution.*
